# Structural analysis of the mutant protein D26G of human γS-crystallin, associated with Coppock cataract

**Published:** 2013-06-05

**Authors:** Srinivasu Karri, Ramesh Babu Kasetti, Venkata Pulla Rao Vendra, Sushil Chandani, Dorairajan Balasubramanian

**Affiliations:** 1 Prof. Brien Holden Eye Research Centre, Hyderabad Eye Research Foundation, L. V. Prasad Eye Institute, Hyderabad, India; 2 Novarus Discoveries Pvt Ltd, Hyderabad, India

## Abstract

**Purpose:**

To analyze the protein structural features responsible for the aggregation properties of the mutant protein D26G human γS-crystallin (HGSC) associated with congenital Coppock-type cataract.

**Methods:**

cDNAs of wild-type (WT) and D26G mutant HGSC were cloned and expressed in *BL21 (DE3) pLysS* cells and the proteins isolated and purified. Their secondary and tertiary structural features, aggregation tendencies, and structural stabilities were compared using spectroscopic (circular dichroism, intrinsic and extrinsic fluorescence), molecular modeling, and dynamics methods.

**Results:**

No difference was observed between the conformational (secondary and tertiary structural) features and aggregation properties between the WT and D26G proteins. The mutant, however, was structurally less stable; it denatured at a slightly lower concentration of the added chemical denaturant (at 2.05 M guanidinium chloride, cf. 2.20 M for the WT) and at a slightly lower temperature (at 70.8 °C, cf. 72.0 °C for the WT). The mutant also self-aggregated more readily (it turned turbid upon standing; at 65 °C, it started precipitating beyond 200 s, while the WT did not, even after 900 s). Molecular modeling showed that the Asp26-Arg84 contact (and the related Arg84–Asn54 interaction) was disturbed in the mutant, making the latter less compact around the mutation site.

**Conclusions:**

The cataract-associated mutant D26G of HGSC is remarkably close to the WT molecule in structural features, with only a microenvironmental change in the packing around the mutation site. This alteration appears sufficient to promote self-aggregation, resulting in peripheral cataract.

## Introduction

The mammalian eye lens is a protein-packed gel, in which the globular cytosolic proteins of the crystallin family constitute the major components, at concentrations as high as more than 400 mg/ml. The distribution of the crystallins within the lens is asymmetric and biphasic [1]. The lens nucleus and cortical regions are particularly rich in βγ-crystallins, and among these, the evolutionarily highly conserved γS-crystallin is expressed abundantly in the cortical regions of the lens [2]. The compact organization of the crystallins within the lens is believed to generate its transparency. Any disturbance—environmental, metabolic, or genetic—that affects this order leads to compromise in lens transparency and opacification, or cataract. We focus here on a genetic mutation in human γS-crystallin associated with congenital cataract in newborn infants.

The crystal structure of the C-terminal domain of human γS-crystallin (HGSC) is known [3] and the detailed solution structure of murine γS-crystallin has been resolved with nuclear magnetic resonance spectroscopy [4]. This crystallin shares a remarkable structural homology, near identity, with the other γ-crystallins, and is folded using four Greek key motifs, each an interlocking set of four β-strands. Two such motifs are in the N-terminal half of the molecule (sequences 1–40 and 42–83, respectively), and two are more in the C-terminal domain (sequences 88–128 and 129–171, respectively [3]). The two domains fold on each other, leading to a compact, stable, and close-packed arrangement. Mutations in the γS-crystallin gene are thus expected to affect the structure of the protein, causing disturbances in intra- and intermolecular packing. Since detailed analysis of the structure of γS-crystallin is thus available, it appears possible to attempt a protein structural rationale of the mutation or a genotype–molecular phenotype–clinical phenotype correlation.

To date, four such cataractogenic mutations in HGSC have been reported. Mutation G18V, associated with cortical cataract [5], has been analyzed by studying the alteration in the structural organization of the protein by Ma et al. [6] and Brubaker et al. [7,8]. The mutation V42M, associated with bilateral dense cataract [9], has been studied recently by our group [10], and we showed how the mutation distorts the Greek key motif, leading to surface exposure of nonpolar residues leading to the formation of light-scattering self-aggregate particles of the mutant protein. The third mutation S39C, associated with microcornea and cataract [11], has yet to be studied from the protein structural point of view, though it appears likely that, with the exposed cysteine residues of the mutants, intermolecular disulfide bonding and aggregation might occur.

We focus here on the fourth reported mutation, D26G, associated with Coppock cataract [12], by cloning, expressing, isolating, and purifying the mutant human γS-crystallin and comparing its properties with those of the normal or wild-type (WT) HGSC. Our results suggest that the mutation causes no significant changes in the molecular architecture of the protein, only local microenvironmental alterations around the mutation site, leading to a relatively less stable molecule, which tends to aggregate upon standing.

## Methods

The methods followed were the same as those described in our earlier papers [10,13]. We describe them briefly below.

### Overexpression, purification, and analysis of the secondary and tertiary structures of the proteins in solution

Wild type and D26G mutant clones were generated as previously described [13]. The sets of primers used for cloning and sequencing of the wild type and D26G mutant are listed in Table 1. The methods followed for the overexpression using pET21-a-γSD26G and BL21(DE3) pLysS cells, and purification of the proteins using SP Sepharose cation-exchange and Sephadex G75 chomatography, followed by Amicon filtration using 3 kDa cut-off membranes, followed by SDS-PAGE and mass spectrometric confirmation of purity, were the same as those described in detail in our earlier papers [10]. As in earlier papers, we used circular dichroism (CD) spectroscopy to monitor the chain conformation, and fluorescence spectroscopy, using intrinsic fluorophores (especially tyrosine residues), whose emission maximum and intensity, as well as the quenching of this fluorescence by quenchers such as KI and acrylamide, according to the procedure in [14], and surface hydrophobicity probes, namely, 9-diethylamino-5H-benzo[alpha]phenoxazine-5-one, or Nile Red [15] and 4,4'-bis (anilinonaphthalene 8-sulfonate), or bis-ANS [16].

**Table 1:** List of primers used for cloning and sequencing HGSC and its D26G mutant.

Clone	Primer	Primer sequence
pET21-a-γS	Forward	5′GGGAGTTCCATATGTCTAAAACTGGAACC3′
	Reverse	5′CCGGAATTCTTACTCCACAATGCG3′
pET21-a-γSD26G	Forward	5′CTATGACTGTGATTGCGGCTGTGCAGATTTCCACACATAC 3′
	Reverse	5′GAAATCTGCACAGCCGCAATCACAGTCATAGCGAC 3′
T7	Forward	5′TAATACGACTCACTATAGG3′
T7	Reverse	5′TATGCTAGTTATTGCTCAG3′
BGH	Reverse	5′TAGAAGGCACAGTCGAGG3′

### Monitoring amyloid-type behavior

The extrinsic fluorescence probe Thioflavin-T (2-[4-(dimethylamino) phenyl]-3,6-dimethyl-1,3-benzothiazol-3-ium chloride) was used to monitor the formation of amyloid-type fibrils [17]. Spectra were recorded between 470 and 570 nm, using an excitation wavelength of 444 nm with 10 nm slit widths. The protein concentrations used were 0.1 mg/ml in buffer 50 mM Tris, pH 7.3. Baselines of the buffer alone were subtracted, and the average of three runs is displayed.

### Chemical and thermal protein unfolding

#### Chemical denaturation

Equilibrium unfolding experiments, using guanidinium chloride (GuCl), were performed at room temperature by diluting the purified proteins to 0.1 mg/ml in a series of solutions ranging in concentrations of 0 to 4.5 M GuCl in a buffer containing 50 mM Tris, 1 mM EDTA, and 5 mM DTT, using Mills et al.’s method [18]. Samples were incubated at 37 °C for 24 h. Fluorescence emission spectra were recorded for each sample using the method described above. Data were analyzed by plotting the concentration of GuCl for each sample versus the ratio of fluorescence intensities at 360 and 320 nm. The protein concentration was 0.1 mg/ml for the unfolding studies.

#### Thermal denaturation study

Here again, fluorescence spectra were used to monitor the unfolding as a function of temperature. Protein solutions were heated continuously from 60 °C to 86 °C, and the fluorescence data were collected every 2 °C after 3 min equilibration at the given temperature. In all fluorescence experiments, the response time used was 0.08 s, the scan speed was 60 nm/s, and the photomultiplier tube voltage was below 400V. Unfolding data were fit to Greene and Pace’s two-state model [19] or Clark et al.’s three-state model [20], using Graphpad Prism software. The model that best fit the data was selected based on a random distribution of residuals. Transition midpoints and ΔG° were calculated for all transitions from these fits. For the time-dependent scattering experiment, the protein solutions were heated at 65°, and scattering was measured at 600 nm as a function of time, up to 900 s.

### Molecular modeling and dynamics study

#### Structural modeling

Human and mouse γS-crystallins share 89.9% identity, and therefore, the solved structure of the murine protein was used as a template for the molecular modeling techniques following established approaches [21,22]. The molecular dynamics simulations were performed using Gromacs 4.5.5, developed by the Berendsen group [23], and run on the Apple Computer operating system OS 10.7. The selection of suitable rotamers of D26, N54, and R84 amino acids were performed in the Discovery Suite (Accelrys Inc., San Diego, CA), as was the generation of the mutant 26G. The Charmm force field [24] terms were used throughout. Visualization of structures was done with InsightII and Discovery (Accelrys Inc.). Initial structures were prepared for simulations by soaking in approximately 21,000 water molecules in a dodecahedral box, with the net charge of the system set to zero by the addition of counterions. After a 2,000-iteration minimization, of the ensemble, the water molecules were equilibrated for 200 ps with harmonic constraints on the protein gradually released. Following this, molecular dynamics simulations were continued for 2 ns at 300 K and with periodic boundary conditions.

## Results

**Figure 1:**
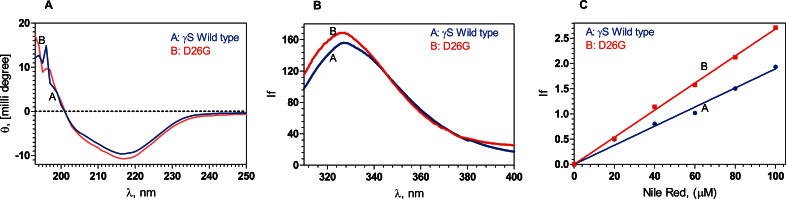
Secondary and tertiary structural analysis of the proteins. **A**: The backbone folding does not alter upon mutation. Estimation of the secondary structures of the proteins, using far-ultraviolet circular dichroism (CD) spectra in the region 195–250 nm, using a JASCO CD spectrometer, at ambient temperature (27 °C), recorded with 2 mm path length cells. **B**: Intrinsic fluorescence of the wild-type and D26G human gamma S-crystallin (HGSC) differ little: Curve **A** (Blue): γS wild-type; **B** (Red): D26G; with excitation wavelength 295 nm and emission wavelength recorded from 300 to 400 nm, at ambient temperature, using a Hitachi spectrofluorimeter. **C**: The mutation has a slightly more set of surface-exposed residues: Extrinsic emission spectra of the surface probe Nile Red. **A** (Blue): γS wild-type; **B** (Red): D26G; extrinsic fluorescence spectra were recorded between 570 and 700 nm with excitation at 540 nm; and slit size 10 nm for excitation and emission. In each set of spectra in **A**, **B**, and **C**, the protein concentrations used were 5 µM (0.1 mg/ml) in 50 mM Tris buffer (pH 7.3), cell path length 3 mm, and spectra recorded at 5.0 nm excitation and emission slits. Spectra shown were the average of three runs.

In Figure 1A, the secondary structural features of WT and D26G HGSC are compared using CD spectroscopy. The near identity of the spectra suggests that no significant changes occurred to the backbone conformation to the protein after the aspartic acid residue was substituted by glycine in the first Greek key motif. Both molecules are folded predominantly in the β-pleated sheet conformation, as shown by the negative band at 218 nm. Figure 1B shows the intrinsic fluorescence due to the tryptophan (and tyrosine) residues in the proteins. Emission in the 327 nm region suggests that the immediate microenvironment around the aromatic side chains is about the same, and the value of 327 nm suggests that they are buried in a nonpolar environment. We next attempted to quench this fluorescence using the ionic quencher KI, which accesses the surface regions of the protein [14]. The Stern-Volmer quenching constant for KI in the wild-type HGSC was estimated to be K_sv_=0.12±0.02, while that for D26G was 0.21±0.04, suggesting that the mutation affects the microenvironment around the aromatic residues to become slightly more accessible. To check this further, we studied the binding of the proteins to the neutral extrinsic fluorescence probe Nile Red, which reports on the surface regions of the macromolecule [15]. Figure 1C shows the emission intensity of Nile Red bound to the mutant is progressively higher than when it is bound to WT; at 100 µM, the probe displays a higher intensity when bound to D26G than to WT (2.75 arbitrary units cf. 1.9). Likewise, when the other surface probe bis-ANS was used [16], it displayed an intensity of 12.5 units when bound to D26G and 10 units when bound to WT (figure not shown).

**Figure 2:**
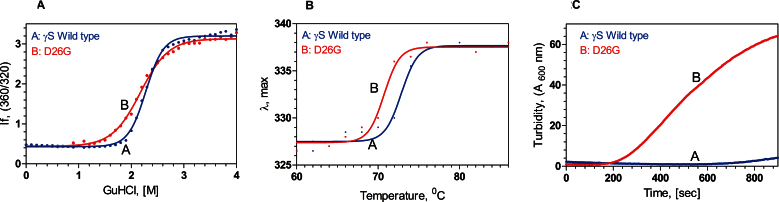
The mutant is structurally weaker and aggregates more readily. **A**: D26G is slightly less stable. Chemical unfolding of human gamma S-crystallin (HGSC; blue) and its mutant D26G (red), monitored using the ratio of the fluorescence intensity at 360 nm (for the unfolded form) and 320 nm (native form), followed using the spectrofluorimeter above. Protein concentrations were 0.1 mg/ml in 50 mM Tris buffer (pH 7.3), with 1 mM EDTA and 5 mM DTT. **B**: The mutant denatures at a lower temperature. Thermal unfolding of HGSC (blue) and D26G (red), monitored by following the change in the wavelength of emission (from 327 nm for the native form to 338 nm for the denatured form), with temperature, using the spectrofluorimeter. Protein concentrations were 100 μg/ml in 50 mM Tris buffer (pH 7.3), with 1 mM EDTA and 5 mM DTT. **C**: D26G self-aggregates. Turbidity or light scattering by wild-type (blue) and D26G HGSC (red) at 600 nm, measured as a function of time, is shown. The temperature was fixed as 65 °C, and A_600 nm_ was followed, using the spectrofluorimeter, using 2.5 nm excitation and emission slits. Protein concentrations were 10 µg/ml, in 50 mM Tris buffer, pH 7.3, with 1 mM EDTA and 5 mM DTT.

We next studied the stability of the proteins using chemical and thermal denaturation methods. We followed the denaturation process by monitoring the change in the ratio of the intrinsic fluorescence of the protein (native molecule monitored at 320 nm and unfolded at 360 nm, per Mills et al. [18]). Figure 2A shows that the wild-type displayed the typical native-to-unfolded two-state behavior when treated with the denaturant GuCl, with the midpoint of transition C_m_ of 2.2 M GuCl and an estimated free energy of denaturation ∆G=8.03±0.34 kcal/m (p<0.001) at the chosen protein and buffer concentrations. The mutant unfolds at a slightly lower denaturant concentration (C_m_=2.05 M GuCl), but the unfolding curve is not as smooth as that of WT, and the curve fitting using the two-state model [19] was less accurate (p=0.12) and gave a value of ∆G=4.8 kcal/m. (Attempts to fit the data using the three-state model [20] with a partially unfolded intermediate proved error-prone.) When the proteins were denatured upon heating (thermal denaturation, followed using intrinsic fluorescence maximum value, Figure 2B), the transition curves for both proteins were quite similar, and essentially two-state in shape. The wild-type showed the midpoint of transition at 72.0 °C, agreeing with Brubaker et al. [7], while D26G denatured at a lower temperature, with the midpoint at 70.8 °C, suggesting that the mutation slightly decreases the stability of the molecule.

We noticed during the thermal denaturation process that although the wild-type molecule was essentially scatter-free even at 80 °C, the mutant began slowly becoming turbid at 65 °C and beyond. We therefore decided to monitor the light scattering of the proteins at the arbitrary temperature of 65 °C, by following the turbidity of the protein solution at 600 nm with time, as was done in [10]. Figure 2C shows that although the WT is essentially scatter-free when monitored for as long as 15 min (900 s), the mutant starts to scatter light by 300 s, and starts to precipitate beyond 900 s. (We also observed that the scattering behavior was concentration-dependent. Although the A_600 nm_ value was 10 arbitrary units, at 300 s of examination, when the mutant protein was studied at 10 μg/ml concentration, it jumped to 75 units when the protein concentration was 100 μg/ml, and to 150 units at 500 μg/ml, at the same time interval of 300 s.) This behavior is similar to what Takata et al. [25,26] reported in the case of the deamidated mutant of human βA3-crystallin, which is associated with congenital cataract.

Structurally disturbed crystallins are known to form amyloid-type aggregates, which bind to the dye Thioflavin T and increase its fluorescence intensity around 490 nm [17]. Titration of the wild-type HGSC with increasing amounts of Thioflavin T (up to a final concentration of 100 μM) raised the emission of the dye from 0 to 6.5 arbitrary units; with the D26G mutant, the increase in the induced emission of the dye through the same concentration range was practically the same as was seen with the wild-type protein (increase from 0 to about 8.5 units at 100 μM dye). Thus, it appears that the mutation does not generate any amyloid-type aggregates.

## Discussion

**Figure 3:**
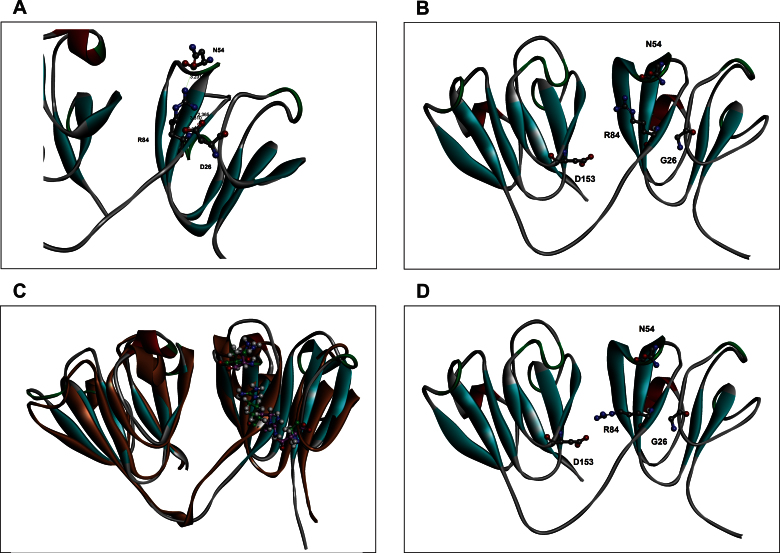
Details of local packing changes caused by replacing D by G in position 26 of the molecule. Molecular modeling of wild-type and D26G, using the methods described in the Methods section. **A**: The interaction between D26 and R84 is shown. The numbers shown in the figure are in Ǻ units. **B**: The effect of replacing D26 by G is shown. **C**: The two structures (mutant in brown and WT in green) are overlain. Panel D shows how R84 in the mutant, having lost contact with D26, might enter into a long distance contact with D153 in the C-terminal domain.

The results suggest that the replacement of the acidic D residue at position 26 in the first Greek key motif of HGSC affects the intramolecular interactions around the mutation site, but without otherwise altering the protein structure. The effect here is not as large as seen in the mutant V42M, where the mutation distorts the motif [10], or even G18V where the bulkier V replaces G in the motif, and appears to expose cysteine residues that might generate intermolecular disulfide aggregates [7,8]. Replacement of D by G changes the van der Waals volume in the site from 91 Å^3^ for Asp to 48 Å ^3^ for glycine, and the accessible surface area from 106 Å^2^ to 0, since glycine has no side chain [27]. To get more insight, we performed molecular modeling and simulations, as shown in Figure 3.

We note that in the model of the wild-type molecule, the anionic side chain of Asp26 interacts with the cationic side chain of residue Arg84 (Figure 3A). In the selected rotameric conformation of R84, Asn54 also lay within interacting distance of Arg84. In structures obtained after 2 ns of molecular dynamics, the wild-type structure exhibited a strong D26-R84 interaction (figure not shown). In the mutant (Figure 3B), the Arg84 side chain moves closer to N54, with the absence of arginine–aspartic acid interaction leading to a widening of the distance between the adjacent antiparallel β-strands that bear these two amino acids. However, the Greek key folds are not lost or distorted in any major manner (see the overlays of WT and D26G in Figure 3C). The initial selection of the arginine rotamer has a strong bearing on the observed interactions, as it is difficult to sample the conformational space of an amino acid with a long side chain. Our examination of possible R84 conformations show that in the G26 mutant, aside from contact with N54, it might enter into a weaker, long-distance contact (4 Å) with the anionic side chain of D153 in the C-terminal domain (Figure 3D).

It thus appears that the mutation alters Coulombic contacts between residues (and alters the isoelectric point of the protein from a pI value of 6.43 for the wild-type to pI=6.89 in D26G [28], causing microenvironmental packing changes around the mutation site, without affecting the overall three-dimensional structure in any major manner). In this respect, mutation D26G, which is associated with Coppock cataract, is somewhat more akin to the mutant G18V (which is associated with progressive cortical cataract) than the mutant V42M (associated with dense cataract), though it does not display any intermediate state during unfolding as G18V does [6-8]. Indeed, the behavior of D26G human γS-crystallin is remarkably similar to what has been reported in the peripheral cataract-associated mutants P23T [29], and R36S and R77S of human γD-crystallin [30,31], where the pI value also changes, but no significant changes occur in the protein architecture or alteration in the protein solubility, excepting microenvironmental changes around the mutation site. Such mutants appear to belong to what has been termed “protein condensation disease,” rather than “protein disorder disease,” which involves alteration of the native state of the molecule. The term protein condensation disease, introduced by Benedek [32,33], refers to situations in which the initial pathological step is the formation of amorphous protein aggregates. The mutation leading to such a condition does not alter the protein chain conformation, only tertiary structural changes that cause net intermolecular interactions leading to aggregation (condensation) and reduction in solubility; an example is the E6V mutation in β-globin, resulting in sickle cell disease. Since the native structure of the wild-type molecule is not altered in a major manner here, it is also referred to by some as “native state aggregation.” This is thus different from “protein disorder disease,” such as ones involving prions, in which the native structure is disturbed.
